# Whole-genome sequencing identifies responders to Pembrolizumab in relapse/refractory natural-killer/T cell lymphoma

**DOI:** 10.1038/s41375-020-1000-0

**Published:** 2020-08-05

**Authors:** Jing Quan Lim, Dachuan Huang, Tiffany Tang, Daryl Tan, Yurike Laurensia, Rou-Jun Peng, Esther Kam Yin Wong, Daryl Ming Zhe Cheah, Burton Kuan Hui Chia, Jabed Iqbal, Nicholas Francis Grigoropoulos, Maarja-Liisa Nairismägi, Cedric Chuan Young Ng, Vikneswari Rajasegaran, Huangming Hong, Seok Jin Kim, Junhun Cho, Eric Tse, Benjamin Mow, Qi-Chun Cai, Li-Mei Poon, Qing-qing Cai, Jing Tan, Jason Yongsheng Chan, Johnathan Xiande Lim, Yeow Tee Goh, Colin Phipps, Olaf Rötzschke, Chee Leong Cheng, Jeslin Chian Hung Ha, Lay Poh Khoo, Yvonne Su Ming Loh, Rex Au-Yeung, Thomas Sau-Yan Chan, Yok-Lam Kwong, William Hwang, Won Seog Kim, Jin-Xin Bei, Tongyu Lin, Choon Kiat Ong, Soon Thye Lim

**Affiliations:** 1 State Key Laboratory of Oncology in South China, Collaborative Innovation Center of Cancer Medicine, Sun Yat-sen University Cancer Center, Guangzhou, 510060 China; 2 Lymphoma Genomic Translational Research Laboratory, Cellular and Molecular Research, National Cancer Centre Singapore, 11 Hospital Drive, Singapore, 169610 Singapore; 3 ONCO-ACP, Duke-NUS Medical School, 8 College Road, Singapore, 169857 Singapore; 4 Division of Medical Oncology, National Cancer Centre Singapore, 11 Hospital Drive, Singapore, 169610 Singapore; 5 Raffles Cancer Centre, Raffles Hospital, 585 North Bridge Road #10-00, Singapore, 188770 Singapore; 6 Department of Haematology, Singapore General Hospital, Outram Road, Singapore, 169608 Singapore; 7 Department of Pathology, Singapore General Hospital, 20 College Road, Academia, 169856 Singapore; 8 Laboratory of Cancer Epigenome, Division of Medical Sciences, National Cancer Centre, 11 Hospital Drive, Singapore, 169610 Singapore; 9 Division of Cancer and Stem Cell Biology, Duke-NUS Medical School, 8 College Road, Singapore, 169857 Singapore; 10 Department of Medical Oncology, Sun Yat-sen University Cancer Center, Guangzhou, 510060 China; 11 Division of Hematology-Oncology, Department of Medicine, Samsung Medical Center, Sungkyunkwan University School of Medicine, Seoul, South Korea; 12 Department of Pathology, Samsung Medical Center, Sungkyunkwan University School of Medicine, Seoul, South Korea; 13 Department of Medicine, The University of Hong Kong, Queen Mary Hospital, Pokfulam, Hong Kong; 14 Mount Elizabeth Medical Centre, Singapore, Singapore; 15 Guangdong Provincial People’s Hospital, Guangdong Academy of Medical Sciences, Guangzhou, China; 16 Department of Haematology-Oncology, National University Cancer Institute of Singapore, National University Health System, Singapore, Singapore; 17 Singapore Immunology Network (SIgN), A*STAR (Agency for Science, Technology and Research), 8A Biomedical Grove, Singapore, 138648 Singapore; 18 Lymphoma Genomic Translational Research Laboratory, Division of Medical Oncology, National Cancer Centre Singapore, 11 Hospital Drive, Singapore, 169610 Singapore; 19 Department of Pathology, The University of Hong Kong, Queen Mary Hospital, Pokfulam, Hong Kong; 20 Director’s office, National Cancer Centre Singapore, Singapore, Singapore; 21 Genome Institute of Singapore, 60 Biopolis Street Genome, Singapore, 138672 Singapore; 22 Duke-NUS Graduate Medical School, 8 College Road, Singapore, 169857 Singapore; 23 Office of Education, Duke-NUS Medical School, Singapore, Singapore

**Keywords:** Cancer genomics, Translational research

## To the Editor:

Antibodies targeting the immune checkpoint axis have been approved by the FDA for the treatment of a broad range of malignancies [1]. NKTCL is an aggressive hematological malignancy derived from NK or T cells with ubiquitous Epstein–Barr virus (EBV) infection, and there is no standard therapeutic option established for patients with relapse/refractory (RR) NKTCL. Although clinical use of immune checkpoint inhibitor (ICI) in RR-NKTCL setting is scarce, two recent case series has achieved a combined complete response rate of 50% [2, 3]. Conversely, a proportion of these patients will be exposed to the side effects and cost of ICI without deriving any clinical benefit. It is therefore crucial to identify robust biomarkers that will reliably identify patients with RR-NKTCL with a high likelihood of response to ICI.

**Fig. 1:**
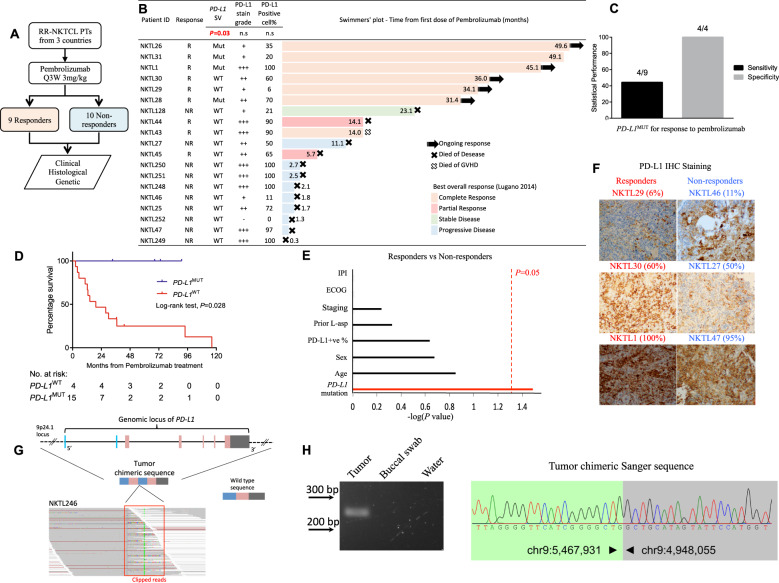
*PD-L1* structural rearrangements (*PD-L1*^MUT^) as a potential biomarker of response to pembrolizumab for patients with RR-NKTCL. **a** Schematic makeup of the study and the stratification of patients with RR-NKTCL accordingly to their response to pembrolizumab. **b** Swimmers’ plot showing the duration of responses for the 19 patients with RR-NKTCL who were treated with pembrolizumab. Tabular data showing the *PD-L1* mutation status, immunohistochemical (IHC) PD-L1 positivity of tumor cells and PD-L1 stain grade (− is negative, + is weakly stained, ++ is moderately stained and + ++ is strongly stained) accompanies each corresponding NKTCL sample. **c** Statistical performance measures of sensitivity and precision by *PD-L1*^MUT^ as a predictor for responders to pembrolizumab. **d** Kaplan–Meier plot comparing the overall survival of patients with *PD-L1*^MUT^ and *PD-L1*^WT^ tumors. **e** Statistical tests on various clinical features and gene-mutation between responders and non-responders were carried out and the respective -log(*P* value) were plotted. The vertical red line denotes the cutoff for significance at *P* = 0.05. **f** Representative images of PD-L1 IHC weakly, moderately, strongly stained images for tumors from both responders and non-responders of patients with RR-NKTCL to pembrolizumab. Percentages of tumor cells positively stained by PD-L1 antibody are in brackets. **g** Schematic diagram of the wild-type 9p24.1 locus and the chimeric sequence representing the *PD-L1*^MUT^ detected in the tumor DNA of NKTL246. A snapshot of the aligned sequencing reads, which are soft-clipped, at the genomic breakpoint of the *PD-L1*^MUT^ are shown in the ‘red’ box. **h** PCR-based gel validation correctly amplified the 246 bp chimeric *PD-L1* sequence from the tumor (T), and not from the buccal swab (BS), water (H_2_0). Sanger sequence validated the chimeric *PD-L1* to base-pair resolution. R responder, NR non-responder, MUT mutant, WT wild type, IPI international prognostic index, ECOG eastern cooperative oncology group, n.s. not significant.

Several clinical biomarkers for ICI in solid malignancies have been reported including programmed death-ligand 1 (PD-L1) expression, tumor mutational burden, T-cell repertoire and human leukocyte antigen class diversity [4]. However, to our knowledge, none of these biomarkers have been able to precisely predict for response in hematological malignancies such as RR-NKTCL, where ICI holds considerable promise [2, 3]. In order to address this gap, we conducted a systematic retrospective clinical, histological, and genetic analysis of 19 patients with RR-NKTCL treated with the ICI pembrolizumab from six medical centers (Fig. 1a). Having identified cryptic rearrangements of the *PD-L1* gene as a strong positive predictor of response to pembrolizumab, we initiated prospective screening of newly relapsed NKTCL cases for this novel biomarker and provided proof of concept for this approach.

The diagnoses of NKTCL in 21 patients in this study were confirmed according to the 2008 World Health Organization classification [5]. Investigations were carried out according to the principles expressed in the Declaration of Helsinki and all patients provided written informed consent.

We performed retrospective genetic profiling on 19 patients and prospective screening on two patients with RR-NKTCL (*N* = 21) from Singapore, Seoul and Hong Kong. In the absence of other viable therapeutic options, the 19 patients who were retrospectively profiled were treated with pembrolizumab. Twenty-one tumors (19 formalin-fixed paraffin-embedded (FFPE) and two snap-frozen) and 14 matching non-tumoral samples were collected for histopathological and genomic sequencing. NKTL1, NKTL25 and NKTL27 were collected at the time of initial diagnosis while the other samples were collected at time of relapse before ICI therapy. All patients provided written informed consents. The list of genes that were targeted by our custom probe-based panel is summarized in Table S1. The list of primers used in the PCR-based validation can be found in Table S2.

The study is designed with a type-I error of P < 0.05, >80% power and to confidently pick up a biomarker with an estimated recurrence of 25% in the pembrolizumab-treated NKTCL cohort with Fisher’s exact test; minimum size of cohort was determined to be 16.

Comparative analyses were done on clinical, histological and genetic features to identify potential biomarkers for response. Fisher-exact tests, Wilcoxon rank-sum tests and log-rank tests were used to test for significance on categorial, discreet-valued and survival analysis between categorial subgroups using Kaplan-Meier statistics, respectively. *P* < 0.05 defines statistical significance in this study. Statistical sensitivity and specificity were determined as per standard methodology [6].

The same pathologist assessed the immunohistochemistry (IHC) PD-L1 positivity of tumor cells centrally for all samples in this study. The same staining procedure U OptiView DAB (3,3’-Diaminobenzidine) IHC v5 was performed on available tumoral samples from the initial retrospective cohort. FFPE tissue sections were cut onto Bond Plus slides. Tissue slides were then subjected to deparaffinisation, rehydration and heat-induced epitope retrieval using a Leica Bond Max autostainer (Leica Biosystems Melbourne). The slides were incubated with the monoclonal PD-L1 antibody (SP263, Ventana, CA, USA). Epstein-Barr encoding region in situ hybridization was used to determine tumoral sections of the stained slides. Images were acquired for each case using a Vectra 3 pathology imaging system microscope (PerkinElmer Inc) at a magnification of 400x.

Additional details on the methods of genomic sequencing and variant-calling are in the Supplementary Notes.

Nineteen patients with RR-NKTCL were treated with pembrolizumab in our group between 2015 and 2019. Each patient was given pembrolizumab at 3 mg/kg about once every three weeks until disease progression or intolerable treatment-related toxicity. As per Lugano 2014 classification [7], complete response, partial response, stable disease and progressive disease were observed in seven (36.8%), two (10.5%), one (5.3%) and nine (47.4%) patients, respectively (Table 1). Overall response rate was 47.4% and among these nine responders, long-term clinical benefit averaging at 28.3 months (95% C.I. [17.4, 39.2] months) was achieved from pembrolizumab treatment (Fig. 1b). The basic clinical features of patients such as age, sex, prior L-asparaginase treatment, Ann Arbor staging, international prognostic index, and performance status did not differ between the responders and non-responders (Tables 1 and S3). The adverse events due to pembrolizumab included severe pneumonia in one patient (NKTL46), and another patient (NKTL128) with pneumonia, increased creatinine and arthritis. Overall, treatment-related toxicity was tolerable.

**Table 1:** Clinical features, prior treatments and responses from pembrolizumab for 19 patients with RR-NKTCL.

Patient ID	Sex	Age at diagnosis	Ann arbor staging	ECOG	IPI	OS (months)	Status as January 2020	PD-L1 positivity	Treatments prior to Pembrolizumab			Pembrolizumab treatment	
									CTx (cycles)	RT	TP	Best Response^a^ (Lugano 2014)	DOR^b^, months
Responders (*n* **=** 9)													
NKTL1	M	49	IV	1	2	73	Alive	100%	GELOX (4), SMILE (5), Romidepsin+Bortezomib (1), BV+Benda (1), Lenalidomide+Dara (1)	Nil	Nil	**CR**: PET/CT	45
NKTL26	M	32	I	1	1	68	Alive	40%	SMILE (2), Vinc+DXM+Lasp (1), GELOX (6)	Yes	Nil	**CR**: PET/CT	49
NKTL28	M	46	IV	3	4	33	Alive	70%	SMILE (2), P-GEMOX (1)	Nil	Nil	**CR**: PET/CT	31
NKTL29	M	48	I	0	0	37	Alive	6%	Ifos+MTX+VP+DXM+Pasp (4)	Nil	Nil	**CR**: PET/CT	34
NKTL30	M	38	IV	3	4	43	Alive	60%	SMILE (5)	Nil	Nil	**CR**: PET/CT	36
NKTL31	M	27	IV	0	5	91	Alive	20%	Lasp+DXM+Vinc+AraC (4), CHOP (2), P-GEMOX (2), DXM+Pasp+mitoxantrone+VP (4) P-GEMOX+VP (2)	Nil	Auto-HSCT with BEAM + Thalidomide	**CR:** CT & MRI	41
NKTL43	M	29	IV	2	3	116	Dead	90%	m-BACOD (4), SIMPLE (5), SMILE (3)	Yes	Nil	**CR**: PET/CT Patient subsequently underwent MUD BMT and died from GVHD.	14
NKTL44	M	66	IV	1	2	37	Dead	90%	SIMPLE (6)	Nil	Nil	**PR**: DOD	3
NKTL45	M	42	IV	1	3	94	Dead	65%	SMILE (6), GEMOX (1)	Nil	Allo-HSCT	**PR**: DOD	2
Non-responders (*n* **=** 10)													
NKTL25	M	30	IV	0	2	14	Dead	72%	SMILE (6), GEMOX (1)	Yes	Allo-HSCT	**PD**: DOD	NA
NKTL27	M	59	IV	0	2	19	Dead	50%	SMILE (3), GIFOX (4)	Nil	Nil	**PD**: DOD	NA
NKTL46	F	62	IV	3	5	12	Dead	12%	CHOP (1), P-GEMOX (1), Thalidomide+Prednisone (1), Lenalidomide+Prednisone (1), Abraxane (1)	Nil	Nil	**PD**: DOD	NA
NKTL47	M	51	IV	1	3	5	Dead	95%	SMILE (1), MILE (2), GIFOX (4)	Nil	Nil	**PD**: DOD	NA
NKTL128	F	62	IV	-	-	31	Dead	21%	GEMOX (6), Chidamide(1)	Nil	Nil	**SD**: DOD	NA
NKTL248	F	79	IV	1	3	10	Dead	100%	VIDL (1), GDP (2), Avelumab (2)	Nil	Nil	**PD**: DOD	NA
NKTL249	M	45	IV	1	2	4	Dead	100%	VIDL (2), GDP (1),	Nil	Nil	**PD**: DOD	NA
NKTL250	M	45	IV	1	2	12	Dead	100%	VIDL (4)	Nil	Auto-HSCT	**PD**: DOD	NA
NKTL251	M	66	I	1	1	28	Dead	100%	Cisplatin (6), Dara (10), GDP (4), Avelumab (3)	Yes	Nil	**PD**: DOD	NA
NKTL252	M	73	IV	2	3	2	Dead	0%	Lasp (2)	Nil	Nil	**PD**: DOD	NA

^a^As assessed by Lugano 2014 criteria: *CR* Complete response, *PR* partial response, *SD* stable disease, *PD* progressive disease. MUD BMT, matched unrelated donor bone marrow transplant; GVHD, graft versus host disease; DOD, died of disease.

^b^DOR: Durability of response was recorded in months from documentation of response until PD as of January 2020.

Abbreviations for treatment regimens: *BV* brentuximab vedotin, *Benda* bendamustine, *Dara* daratumumab, *Vinc* vincristine, *DXM* dexamethasone, *Lasp* L-asparaginase, *Ifos* ifosfamide, *MTX* methotrexate, *VP* etoposide, *Pasp* Pegaspargase, *AraC* cytarabine, *ND* not done, *VIDL* etoposide, ifosfamide, dexamethasone, and L-asparaginase, *GDP* gemcitabine, dexamethasone, cisplatin, *P-GEMOX* Pegaspargase, gemcitabine, and oxaliplatin, SMILE, Dexamethasone, methotrexate, ifosfamide, L-asparaginase, and etoposide; *CHOP* cyclophosphamide, doxorubicin, vincristine, prednisone, *GIFOX* Gemcitabine, ifosfamide, oxaliplatin and rituximab, *SIMPLE*, Cisplatin, Gemcitabine, Ifosfamide, Etoposide, L-asparaginase and Dexamethasone.

*ECOG* eastern cooperative oncology group, *IPI* international prognostic index, *OS* overall survival, *RT* radiotherapy, *TP* transplant.

To investigate if there exist genomic alterations that could be enriched within the responders, we performed next-generation sequencing on 19 pre-pembrolizumab RR-NKTCL samples and 13 matched normal tissues. Strikingly, the most frequent somatic mutations were structural rearrangements disrupting the 3’-UTR of *PD-L1* (*PD-L1*^MUT^) in four cases (21.1%) (Fig. S1). Frequent *PD-L1* structural rearrangements was first reported in adult T-cell Leukemia/Lymphoma [8], but its effect on response to ICI therapy in the clinical setting is unclear [9]. Importantly, *PD-L1*^MUT^ was the only gene alteration that was significantly enriched in the tumoral tissues of patients who responded to pembrolizumab compared to those who did not (*P* = 0.03, Fisher’s exact test) (Fig. 1b and Table S4). In fact, the four patients’ tumors that are *PD-L1*^MUT^ responded to pembrolizumab and none of the 10 tumors from the non-responders harbored this variant. Consequently, in our cohort, *PD-L1*^MUT^ achieved 100% specificity in identifying responders to pembrolizumab. Sensitivity was modest at 44.4% (4/9; *PD-L1*^MUT^ responders / Total responders) (Fig. 1c). In terms of survival outcomes, *PD-L1*^MUT^ cases had significantly better overall survival (Fig. 1d, Hazard ratio=2.97e-09, mean=5.55, 95% C.I. [3.84, 7.26] years, *P* = 0.0279, log-rank test, see also Table S5) than *PD-L1*^WT^ cases (mean = 2.59, 95% C.I. [1.25, 3.93] years) when treated with pembrolizumab. Analysis of traditional clinical predictors of response to standard chemotherapy revealed no significant association with response to pembrolizumab (Fig. 1e and Table S3).

Currently, IHC PD-L1 expression has been validated by clinical Phase III trials as predictive biomarkers for ICI therapy in selected solid malignancies [10]. We found that PD-L1 was expressed in almost all our tumoral specimens (18/19 cases), which is consistent with previous studies [2, 3, 11]. Furthermore, PD-L1 positivity showed large inter-patient variability regardless of whether patients responded (inter-patient range: 6–100%) or not responded (inter-patient range: 0–100%) (Fig. 1f and Table S6). These results clearly showed that PD-L1 positivity is less ideal than *PD-L1*^MUT^ as a biomarker for response to ICI therapy in NKTCL.

To test our hypothesis of using *PD-L1*^MUT^ as a *bona fide* predictor of response to anti-PD-1 therapy, we prospectively screened patients with RR-NKTCL for *PD-L1*^MUT^. Two cases were screened, and one was found to be *PD-L1*^MUT^. The *PD-L1*^MUT^ case was a 70-year-old man who relapsed after first-line treatment with GELOX (gemcitabine, oxaliplatin and L-asparaginase) (Fig. S2). Positron emission tomography/Computed tomography showed disseminated disease involving multiple extra-nodal sites (Fig. S3A). In view of the patient’s high-risk features (advanced age, stage IV, >1 extra-nodal sites and elevated serum lactate dehydrogenase), the patient was not eligible for intensive combined chemotherapy or clinical trial. We sequenced his relapse tumor and buccal swab samples, detected (Fig. 1g) and validated the presence of the somatic *PD-L1*^MUT^ in his tumoral tissue (Fig. 1h). The patient was started on pembrolizumab at a dose of 3 mg/kg every 3 weeks and achieved metabolic CR after the third cycle of treatment (Fig. S3B) despite harboring several high-risk features portending a grim prognosis. As of 30th Jan 2020, he is still in clinical and molecular remission with an undetectable EBV titre (Table S7).

PD-1 blockade has been a promising therapeutic option for NKTCL [2, 3], and this was corroborated by the overall response rate (47.4%, 9/19) observed in our initial retrospective pembrolizumab-treated cohort. NKTCL has been associated with ubiquitous EBV infection and, *HLA-DPB1*, *HLA-DRB1* and *IL18RAP* polymorphisms, suggesting the involvement of immune evasion in its tumorigenesis [12, 13]. EBV is mostly presented as a clonal episomal form with type II latency (EBNA1+, EBNA2-, and LMP1+) in NKTCL [5]. Indeed, almost all of our biopsies from the NKTCL tumors (94.7%, 18/19; Table S6) were positive for membranous PD-L1 which is consistent with the observation that LMP1 induced the expression of *PD-L1* in NKTCL [14]. Conceivably, LMP1-induced PD-L1 could be transiently blocked by pembrolizumab. However, it has been reported that induced PD-L1 is likely a factor of resistance to immune checkpoint blockade as compared to constitutive PD-L1 expression by genetic alterations, such as *PD-L1*^MUT^ that are endogenous within the tumor cells [15]. This could partially explain why some of our patients with *PD-L1*^WT^ but PD-L1+NKTCL did not achieve clinical benefit from pembrolizumab. This highlights the potential of *PD-L1*^MUT^ as a biomarker to select patients with NKTCL for PD-1 blockade therapy.

In conclusion, this is the first study reporting the significant association of *PD-L1*^MUT^ with response to pembrolizumab in patients with RR-NKTCL and tested its clinical usefulness in a prospective case study. Our results showed that *PD-L1*^MUT^ is a potential biomarker to better select patients with NKTCL for anti-PD-1 therapy, improving the cost-economics and minimising adverse events for our patients to ICI therapy.

## Supplementary information

Supplementary Methods and Supplementary Figures

Table S1

Table S2

Table S3

Table S4

Table S5

Table S6

Table S7

## Data Availability

The datasets generated and/or analysed during the current study are uploaded to the European Genome-phenome Archive (EGA) repository with accession EGAD00001004140.

## References

[1] Wei SC, Duffy CR, Allison JP (2018). Fundamental mechanisms of immune checkpoint blockade therapy. Cancer Discov..

[2] Kwong YL, Chan TSY, Tan D, Kim SJ, Poon LM, Mow B (2017). PD1 blockade with pembrolizumab is highly effective in relapsed or refractory NK/T-cell lymphoma failing l-asparaginase. Blood..

[3] Li X, Cheng Y, Zhang M, Yan J, Li L, Fu X (2018). Activity of pembrolizumab in relapsed/refractory NK/T-cell lymphoma. J Hematol Oncol..

[4] Havel JJ, Chowell D, Chan TA (2019). The evolving landscape of biomarkers for checkpoint inhibitor immunotherapy. Nat Rev Cancer..

[5] Chan JKC, Quintanilla-Martinez L, Ferry JA. Peh S-C Extranodal NK/T-cell lymphoma, nasal type. In: Swerdlow SH, Campo E, Harris NL, Jaffe ES, Pileri SA, Stein H, et al., editors. WHO classification of tumours of haematopoietic and lymphoid tissues. 4th ed. Lyon: IARC Press; 2008. p. 285–8.

[6] Yerushalmy J (1947). Statistical problems in assessing methods of medical diagnosis, with special reference to X-ray techniques. Public Health Rep. (1896-1970).

[7] Cheson BD, Fisher RI, Barrington SF, Cavalli F, Schwartz LH, Zucca E (2014). Recommendations for initial evaluation, staging, and response assessment of Hodgkin and non-Hodgkin lymphoma: the Lugano classification. J Clin Oncol..

[8] Kataoka K, Shiraishi Y, Takeda Y, Sakata S, Matsumoto M, Nagano S (2016). Aberrant PD-L1 expression through 3’-UTR disruption in multiple cancers. Nature..

[9] Ratner L, Waldmann TA, Janakiram M, Brammer JE (2018). Rapid progression of adult T-cell leukemia–lymphoma after PD-1 inhibitor therapy. N Engl J Med..

[10] Darvin P, Toor SM, Sasidharan Nair V, Elkord E (2018). Immune checkpoint inhibitors: recent progress and potential biomarkers. Exp Mol Med.

[11] Song TL, Nairismägi M-L, Laurensia Y, Lim J-Q, Tan J, Li Z-M, et al. Oncogenic activation of STAT3 pathway drives PD-L1 expression in natural killer/T cell lymphoma. Blood. 2018. 10.1182/blood-2018-01-829424. 10.1182/blood-2018-01-829424 PMC6148343 30054295

[12] Li Z, Xia Y, Feng LN, Chen JR, Li HM, Cui J (2016). Genetic risk of extranodal natural killer T-cell lymphoma: a genome-wide association study. Lancet Oncol..

[13] Lin G-W, Xu C, Chen K, Huang H-Q, Chen J, Song B (2020). Genetic risk of extranodal natural killer T-cell lymphoma: a genome-wide association study in multiple populations. Lancet Oncol..

[14] Bi XW, Wang H, Zhang WW, Wang JH, Liu WJ, Xia ZJ (2016). PD-L1 is upregulated by EBV-driven LMP1 through NF-kappaB pathway and correlates with poor prognosis in natural killer/T-cell lymphoma. J Hematol Oncol.

[15] Ribas A, Hu-Lieskovan S (2016). What does PD-L1 positive or negative mean?. J Exp Med..

