# Supplementary material for: Whole-genome sequencing identifies responders to Pembrolizumab in relapse/refractory natural-killer/T cell lymphoma
Source: Leukemia. 2020 Aug 5;34(12):3413–9. doi: 10.1038/s41375-020-1000-0 (PMC7685978; doi:10.1038/s41375-020-1000-0)
Supplement: Supplementary file 1 — Supplementary Methods and Supplementary Figures [file 41375_2020_1000_MOESM1_ESM.docx]

Appendix

# Additional information – Methods

## Whole-genome Sequencing

Genomic DNA from tumor tissue was extracted with QIAamp DNA Mini Kit. The DNA for the matching normal was obtained from blood or buccal swabs and purified by Blood and Cell Culture DNA Mini kit or E.Z.N.A. Tissue DNA Kit (Omega Bio-tek) according to manufacturer’s instructions. The quantity and quality were assessed by Quant-iT PicoGreen dsDNA Assay Kit (Invitrogen) and agarose gel electrophoresis. Whole-genome sequencing (WGS) was performed for 13 pairs of tumor-normal samples (Sample ID suffixed by number <100) described in this study. All sequencing libraries were prepared using TruSeq Nano DNA Library Prep Kit (Illumina). Paired-end sequencing was performed on Illumina HiSeq 2000 or HiSeq X Ten as 2x101 bp or 2x151 bp, respectively.

## Targeted-capture Sequencing

Genomic DNA from seven NKTCL formalin-fixed paraffin-embedded (FFPE) specimens was screened for somatic mutations using deep-targeted capture sequencing FFPE rolls or slides were extracted using QIAamp DNA FFPE Tissue kit (QIAGEN). Their samples IDs are suffixed by number ≥100. The FFPE genomic DNA was then treated with NEBNext FFPE DNA Repair Mix and assessed by Quant-it PicoGreen dsDNA Assay Kit (Invitrogen). The library was generated from 10-200 ng DNA with SureSelect^XT^ Low Input Target Enrichment System for Illumina Paired-End Sequencing Library (Agilent Technologies) according to manufacturer’s instructions. RNA based probe was designed with SureDesign (Agilent Technologies) to target-capture 40 genes (Table S1). Next, the captured libraries were pooled in equimolar concentration and sequenced on Illumina Novaseq 6000 platform with SP or S1 chip.

## Sanger Sequencing

Details about PCR conditions and sequencing are described elsewhere.(1) Primers were designed using Primer3 software (2) and the sequences are listed in Table S2. Sanger sequences were aligned back to the reference genome using BLAT (3).

## DNA sequencing read processing and somatic variant-calling

DNA sequencing adaptors, trailing low-quality bases and polyG sequences were removed by fastp (v0.19.6) (4). The DNA sequencing reads were aligned using BWA-MEM (v0.7.17) (5) to the hs37d5 reference genome. Short-variants were called using Strelka2 (v2.9.4) (6) and annotated by wAnnovar (7). The filtering criteria on the short-variants called from tumor-only samples were described in Song *et al* (8). Structural rearrangements were called using Manta (9), annotated by annotSV (v1.2) (10) and validated by Sanger sequencing (Fig S1 and Table S2).

## Supporting Figures


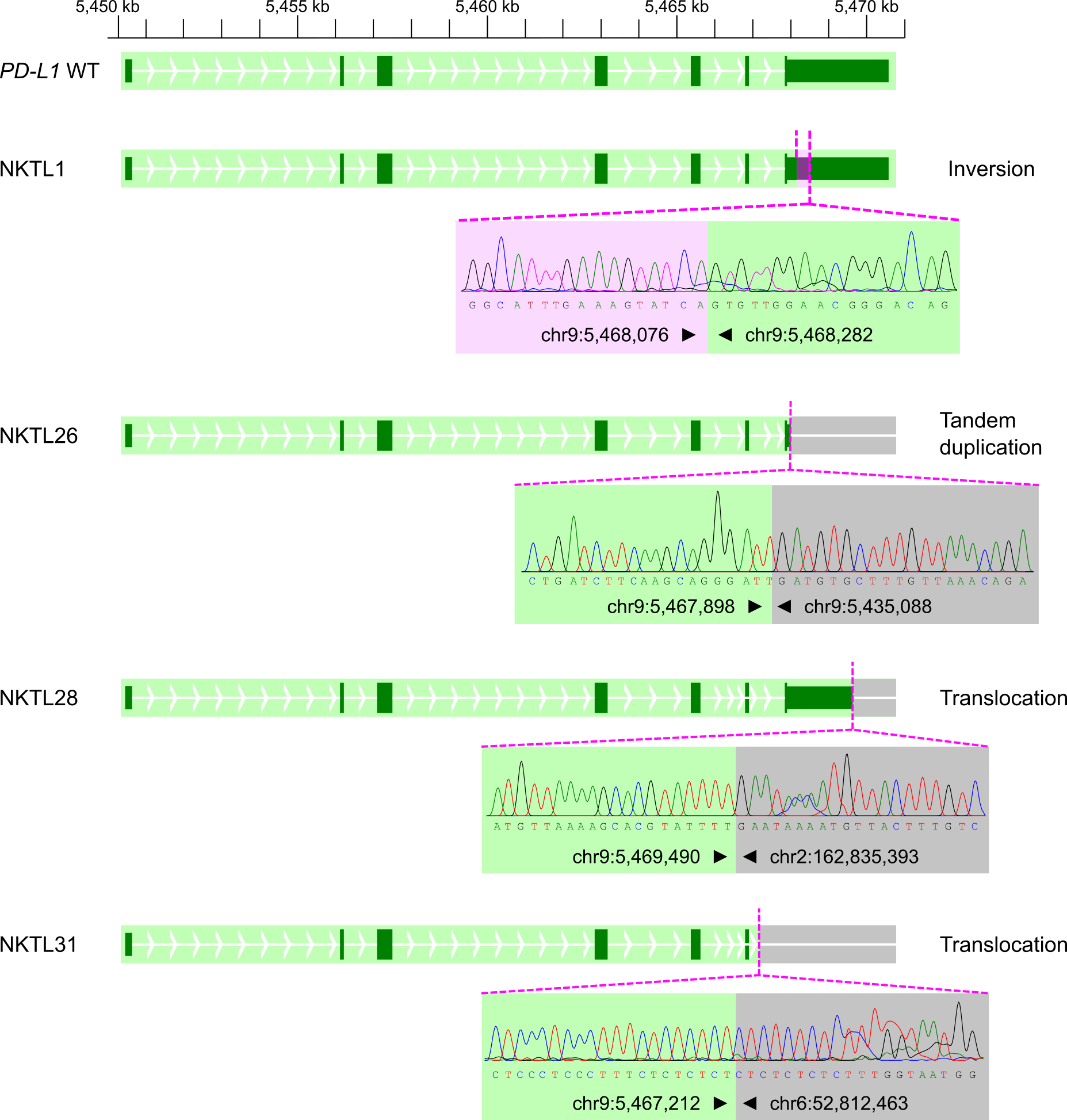


**Fig S1.** Validation of the *PD-L1* rearrangements detected from the sequencing data of 19 Natural-Killer/T-Cell lymphoma tumors. Among the *PD-L1* rearrangements, two are interchromosomal translocations, one is tandem duplication and one is of inversion type. Sanger sequencing was used to confirm the breakpoints (in magenta broken lines) of each *PD-L1* rearrangement identified in the study cohort. The gene structures of the wild type (WT) portions of the *PD-L1* gene are colored in green. Gray background demonstrates fusion to intergenic sequence and purple background demonstrates an inversion from the wild-type DNA sequence. White arrows represent the orientation of transcription.

**Fig S2.** Clinical timeline of patient NKTL246. The top timeline shows the treatments and responses that the patient has experienced from the treatment given to treat his initial and relapse tumors. The bottom line plot shows the corresponding EBV loads throughout his treatment.

A

BA

**Fig S3.** PET/CT scans on the lesions presented of patient NKTL246 before and after pembrolizumab. (A) PET/CT scans on the lesions presented at the time of relapse. (B) PET/CT scans at the nearest-approximate corresponding bodily locations at the time of complete metabolic response to pembrolizumab. White arrows point to the area of scans of SUVmax values. SUVmax: maximum standardized uptake value

# References

1. Nairismagi ML, Tan J, Lim JQ, Nagarajan S, Ng CC, Rajasegaran V, et al. JAK-STAT and G-protein-coupled receptor signaling pathways are frequently altered in epitheliotropic intestinal T-cell lymphoma. Leukemia. 2016;30(6):1311-9.

2. Rozen S, Skaletsky H. Primer3 on the WWW for general users and for biologist programmers. Methods in molecular biology. 2000;132:365-86.

3. Kent WJ. BLAT--the BLAST-like alignment tool. Genome research. 2002;12(4):656-64.

4. Chen S, Zhou Y, Chen Y, Gu J. fastp: an ultra-fast all-in-one FASTQ preprocessor. Bioinformatics. 2018;34(17):i884-i90.

5. Li H. Aligning sequence reads, clone sequences and assembly contigs with BWA-MEM. q-bioGN. 2013:arXiv:1303.3997v1.

6. Kim S, Scheffler K, Halpern AL, Bekritsky MA, Noh E, Kallberg M, et al. Strelka2: fast and accurate calling of germline and somatic variants. Nature methods. 2018;15(8):591-4.

7. Chang X, Wang K. wANNOVAR: annotating genetic variants for personal genomes via the web. J Med Genet. 2012;49(7):433-6.

8. Song TL, Nairismägi M-L, Laurensia Y, Lim J-Q, Tan J, Li Z-M, et al. Oncogenic activation of STAT3 pathway drives PD-L1 expression in natural killer/T cell lymphoma. Blood. 2018:blood-2018-01-829424.

9. Chen X, Schulz-Trieglaff O, Shaw R, Barnes B, Schlesinger F, Kallberg M, et al. Manta: rapid detection of structural variants and indels for germline and cancer sequencing applications. Bioinformatics. 2016;32(8):1220-2.

10. Geoffroy V, Herenger Y, Kress A, Stoetzel C, Piton A, Dollfus H, et al. AnnotSV: an integrated tool for structural variations annotation. Bioinformatics. 2018;34(20):3572-4.
